# Understanding of research: a Sri Lankan perspective

**DOI:** 10.1186/1472-6939-11-7

**Published:** 2010-04-27

**Authors:** Athula Sumathipala, Sisira Siribaddana, Suwin Hewage, Manura Lekamwattage, Manjula Athukorale, Chesmal Siriwardhana, Kumudu Munasinghe, Kethakie Sumathipala, Joanna Murray, Martin Prince

**Affiliations:** 1 Institute for Research & Development, Colombo, Sri Lanka; 2 Institute of Psychiatry, Kings College London, UK; 3 Department of Primary Care and Public Health Sciences, Kings College London, UK

## Abstract

**Background:**

Lack of proper understanding on the part of researchers about public understanding of research and informed consent will increase the potential for malpractice. As a part of a larger study on ethics and informed consent in Sri Lanka, this study aimed to ascertain the level of understanding of 'research' by exploring the views of the public and professionals.

**Methods:**

Convenience sampling and snow ball technique were used for recruitment with an emphasis on balanced age and gender representation, diverse educational, socio-cultural and professional backgrounds, and previous research experience, either as researchers or participants. Content analysis of the data was carried out.

**Results:**

66 persons (37 males, 29 females) participated. Although fundamentally a qualitative study, themes were also quantitatively analysed for informative results. Most participants thought that the word 'research' meant searching, looking, inquiring while some others thought it meant gathering information, gaining knowledge and learning.

A third of participants did not offer an alternative word for research. Others suggested the words survey, exploration, search, experiment, discovery, invention and study as being synonymous. Doctors, health professionals, health institutions, scientists, professionals, businessmen, pharmaceutical companies, students, teachers were identified as people who conduct research.

Participants indicated that crucial information on deciding to participate in research included objectives of the research, project importance and relevance, potential benefits to individuals and society, credibility & legitimacy of researchers, what is expected of participant, reason for selection, expected outcome, confidentiality and ability to withdraw at any time. A majority (89%) expressed their willingness to participate in future research.

**Conclusions:**

The results show that with or without prior experience in research, participants in this study had a reasonable understanding of research. The findings show that a decision about taking part in research is dependent on knowledge, education and also on social networks.

The results demonstrate that the majority were supportive of health research and believe that research is beneficial to the welfare of society.

## Background

The international research community has debated and promoted the discussion on the issue of informed consent in human subject research [1-3]. However, a considerable gap in research literacy still exists between research participants and investigators [4]. This gap along with the authoritative position held by academics may affect the quality of informed consent as much as the desire to participate in research [3]. A lack of proper understanding on the part of researchers about what the public understand by research and consent processes will only widen this gap, increasing the potential for malpractice in conducting research and intentional or unintentional exploitation of vulnerable subjects, especially in the developing world [3,5,6].

Public understanding of research may vary according to levels of education and literacy, the existence of a research culture and the extent of debate about science and research in the public domain, while the value placed on individual informed consent practices may have cultural variations [2,6,7].

Although the understanding and attitudes of patients from Western countries towards research have been investigated [8-10], the perspectives of individuals from developing countries have received little attention [11-13]. An Egyptian study of individuals' attitudes towards medical research reported that although the participants recognized the value and expressed a great deal of trust in medical research, they nonetheless mentioned concerns about the level of risks associated with several types of medical research [11,14]. This study further concluded that participants experienced difficulty in understanding several research concepts: randomization, double-blind, and clinical equipoise. Trust in the physicians conducting research was important in making the decision to participate in clinical research [14]. A recent qualitative study conducted in Malawi concludes that people make rational decisions to participate in research, especially in settings with inadequate health services [15]. This study questions 'therapeutic misconception' in certain low resource settings. A number of studies have been conducted in African countries investigating the attitudes and understanding of research participation, mainly in malaria treatment and vaccine trials [16].

Several guidelines currently exist for guiding health research in both developed and developing country settings [17,18]. But these guidelines do not address the critical issue of public understanding of research, especially in the developing country context where low literacy, religious beliefs and lack of resources dictate the public perception towards health research. The current study was designed to address the lack of empirical evidence on understanding of medical research and informed consent in Sri Lanka. The study focused on individuals' willingness to participate in research in particular and on information they require in order to make a decision to participate.

### Ethical approval

Ethical approval for the project was obtained from ethics review committee (ERC) of the Faculty of Medicine, Sri Jayewardenepura University, Sri Lanka and ERC of the Institute of Psychiatry, King's College London.

## Methods

### Objectives

This was part of a larger study on informed consent in Sri Lanka [19,20]. The aim of this component of the study was to ascertain the level of understanding of 'research' with a particular emphasis on health research and the concept of voluntary informed consent by exploring the views of the general public and a selected group of professionals in Sri Lanka.

### Protocol development process

Along with other components of the project, the initial protocol was subjected to revision based on comments received from reviewers for the funding body. There was also a consultation meeting held in UK to finalise the protocol. The proposal was also presented to an invited audience in Sri Lanka who were either involved in ethics review process or who had a special interest in ethics, some of whom had received training during an intensive course in bioethics funded by the Wellcome Trust in 2003 [19,20].

Following a two-day workshop on qualitative research methods, focus group meetings on the protocol were held to develop consensus. Three rounds of meetings were conducted where strategies for recruiting participants for the study were discussed in depth. Focus group participants felt that it was important to include people who had participated in research as well as people with no research participation experience. Strategies discussed to recruit participants with previous research experience include;

1. Obtain permission from ethics committees to have access to people who participated in previous research.

2. Try to recruit them through researchers who have carried out research projects recently.

3. Door knocking, snowballing technique and convenience sampling

While advantages and disadvantages in all these methods were identified, there were specific ethical concerns arising from using the first and second approaches.

The group discussions led to a final decision to use convenience sampling [21], recruiting through recommendation from the initial participants and using snowballing technique. Purposeful sampling allowed participants with different professional or educational backgrounds to be recruited ensuring diversity of characteristics.

### Sample recruitment

Recruitment was carried out with an emphasis on balanced age and gender representation, diverse educational, socio-cultural and professional backgrounds and on those with previous research experience, either as researchers or participants.

Participants were initially given a verbal description of the study aims and what would be required of them and then invited to participate. An information leaflet and consent form was also provided.

### Data collection

The interview schedule consisted of open-ended questions designed to elicit a wide range of views from the participants (appendix). The original questions were composed in English and translated to Sinhalese to facilitate the data collection. At the beginning of each interview, the interviewer informed the participant that this study was focused on healthcare research. Topics included: 'understanding of research, who does research, whether it is good or bad to do research, who benefits, experience in and willingness to participate in research, what sort of information would they want to know before deciding'.

Three research assistants (SH, ML, MA) conducted the interviews. As in other qualitative work carried out by our team in Sri Lanka, the interviews were not audio recorded as this was felt to be culturally too sensitive and could adversely affect the study participation [22]. Therefore, participants' responses were written verbatim by the interviewers.

A predefined check-list of information important in making a decision to participate in research was included and participants were asked to respond as 'Yes', 'No' or 'Not sure'.

The interviews were conducted with medical students, doctors, teachers, researchers and patients who were selected at Out Patient Departments (OPD) of GP and hospital clinics. Some had previously participated in or conducted research.

### Data analysis

The answers to the open ended questionnaire were recorded in written form in Sinhala and subsequently entered in to a SPSS database by two researchers (ML, MA). Then these were manually coded and entered into separate databases for each question (SH, CS). As the interviews were conducted in Sinhalese, the researchers (SH, CS) jointly translated the answers to English before database entry was conducted.

Content analysis of the data was carried out by reading the transcripts line-by-line analysing the contents [23]. Therefore, interview data under pre determined questions and headings were perused for recurring themes and line by line analysis to identify similar themes. Responses containing similar themes were grouped together and categorised. In order to verify the analysis it was repeated once again by KM and KS. SPSS was also used to analyse the quantitative data presented here.

## Results

### Participant characteristics

A total of 66 persons (37 males; 56%, 29 female; 44%) participated. The mean age of participants was 41 (range 15-72). The age of nine participants was missing.

The sample included 30 (45%) who had participated in research projects previously and 36 who had not. Participants were patients in the government sector and GP setting, researchers (medical and non-medical), research assistants, medical students and teachers including eight teacher-researchers from National Institute of Education (Table 1).

**Table 1:** Study participant representation.

Participants	Number
Previous research participants	7
Researchers	8
Research assistants	4
OPD patients in a hospital	13
OPD patients in a GP clinic	5
Teachers	14
Doctors who have done research	4
Doctors that have not done research	4
Medical students	7

The patients from government hospitals and GP setting were considered to be lay-persons. Their educational or professional backgrounds were not recorded explicitly as it was considered non-relevant with concern to the particular study and setting. Therefore, they were simply categorised as lay patients. Researchers (medical and non-medical) were categorised according to their profession, derived from what they indicated as their profession. Research assistants were also taken as a whole, without differentiation into categories such as health or other fields. All the people categorised as students were in fact medical students from various medical faculties in the country.

### Initial preliminary analyses of data

As there were two main categories of participants in the study; professionals and non-professionals as well as those who had participated in and conducted research, we compared the responses to following three questions to identify differences in understanding or perceptions between the groups.

1. What do you understand by the word "Research"?

2. Are there words similar in meaning to the word "Research"?

3. To your knowledge, what sort of people conduct research?

As there were no observable differences between the groups on these key questions, we analysed the data for the sample as a whole.

Although this is fundamentally a qualitative study we have presented the frequencies for each theme as combined qualitative and quantitative methods are now becoming more popular and informative [24]. These themes were derived and defined from participant responses according to similarities in meanings. The responses were coded according to themes which emerged and subsequently quantified in order to identify frequencies. The similarities were identified originally from the Sinhala language responses, and verified for context and concept after translation in to English.

Because the questions were short and structured, the answers were also short and it was possible to accurately record verbatim without limiting the pace of the interview. For each question most patients provided single worded replies while only a few elaborated their answers. Many participants provided several short answers for some questions, giving different meaning words which encompassed the general idea of the answer. In the analysis, these answers were quantified as separate, ignoring the fact that this may lead to the percentages exceeding 100% in numerical sense. Also these separate answers were given due to the complexities in the Sinhala language, in which this questionnaire was administered. Participants used different words to describe and answer a single concept, but these words have distinct contextual differences, leading to the separate coding done in the study.

### Research Concept

Out of the total 66 participants, 21 (31.8%) did not provide an answer to this question. The responses from the rest are shown in Table 2.

**Table 2:** Concept of research.

**Research**	Searching/look for/inquiries (48.5%) discovery and finding (33.3%) Obtaining data/gathering information/getting to know/gaining knowledge and learning (22.7%) Searching about problems/looking in to problems (13.6%) Testing of blood, urine, stools and other types of laboratory tests ordered by doctors(4.5%)	'Making a judgment about something' 'Experimenting' 'A process that looks in to see if something is correct or not, in a way that is encouraging to the public' 'Coming to a decision by obtaining data using a certain methodology to make something clear' 'An in-depth search' 'Obtaining a description' 'A study to identify a disease' 'Looking in to something to see if it is good or bad' 'Looking in to something, to comprehend something that is not understood, to improve an existing thing' 'Further exploration of something unknown' 'Using statistical knowledge to collect observations, to summarise, to analyse, to present, to come to conclusions in a scientific background and to forward them to those who require them for future progress' 'Developing a hypothesis and searching if it is correct or not'

Total number of participants -- 66

Number of participants responded for this question -- 45

### Synonyms

A third of participants (22; 33.3%) did not offer an alternative word for research. The responses of the other participants are shown in Table 3.

**Table 3:** Words similar in meaning to the word 'research'.

Theme (meaning in Sinhala language)	Number
Survey/Scientific Survey (SAMEEKSHANAYA)	15 (23.%)
Exploration/Scientific Exploration (GAVESHANAYA)	8 (12%)
Search/Scientific Search/Methodical Search (SEVEEMA/SOYA BELEEMA/SODISI KIREEMA)	7 (11%)
Experiment (ATHHADA BELEEMA)	6 (9%)
Test/Examination (PAREEKSHANAYA/PAREEKSHA KIREEMA/PIRIKSUMA)	4 (6%)
Discovery/Invention (SOYA GENEEMA)	3 (5%)
Observation (NIREEKSHANAYA)	3 (5%)
Research (in English)	3 (5%)
Study (ADYAYANAYA)	2 (3%)
Inquiry (VIMASEEMA/VIMARSHANAYA)	2 (3.%)
Manufacturing (NISHPADNAYA)	1 (2%)
Project (VIYAPRUTHIYA)	1 (2%)
Education (ADYAPANAYA)	1 (2%)
Data collection (DHATHTHA EK RAS KEPEEMA)	1 (2%)
Making a judgement (VINISHCHAYA KIREEMA)	1 (2%)

Total number of participants -- 66

Number of participants responded for this question - 44

### People who conduct research

Table 4 shows the types of people who conduct research as indicated by participants.

**Table 4:** What sort of people conducts 'research'?

Doctors, allied medical professionals, health care workers & health institutions	54(82%)
Scientists professionals & learned people	27 (41%)
Businessmen, pharmaceutical & other companies	10(15%)
Anyone	10(15%)
Students & teachers	8 (12%)
Government institutions	8 (12%)
Others	6 (9%)
Nongovernmental organisations (NGO)	5 (8%)

### The purpose of health research

Twenty five (37.9%) said health research is carried out in order to *improve health services to the public, society *and *to find solutions to health problems *in order to make a healthy population. The full set of themes and responses are shown in Table 5.

**Table 5:** Purpose of health research.

Concept	Themes	Supporting Quotes
**The purpose of health research**	Improve health services to the public, society and to find solutions to health problems (37.9%) Search for/discover/introduce/improve and assess the quality of drugs, treatment and cures (25.8%) Search for, broaden and consolidate knowledge, curiosity and for the advancement of science (19.7%) Achieve individual betterment and to meet educational requirements (7.6%), Commercial gain (6.1%)	'To keep people informed' 'To identify new diseases, To look in to the knowledge of the public about diseases' 'For the development of the medical profession of Sri Lanka' 'For exams, 'To make new discoveries those are beneficial to the public as drugs and treatments' 'To consolidate knowledge that has been already discovered' 'To get to know about the health status of the population of the country' 'To relieve their suspicions and curiosities, to make a new discovery' 'To scientifically identify health problems, To broaden your knowledge in the field, For individual betterment' 'For individual educational requirements, Institutional requirements, Interest in the problem'

### Benefits and risks of research

Although all participants expressed the view that conducting research was beneficial, the majority (45; 68.2%), believed that untoward effects may arise from research. Table 6 shows the full results.

**Table 6:** Benefits and risks of research.

Concept	Themes	Supporting Quotes
**Benefits and risks of research**	Benefits Ability to find new knowledge about diseases, new treatment methods for diseases Research helps improve existing knowledge on disease and treatments Research helps to increase productivity of the health sector Risks Adverse effects from participating in research causing harm or death such as drug trials (35.5%) Methodological errors in research resulting in false conclusions (17.8%)	Benefits 'To prove something with scientific evidence as in mosquito eradication' 'To find data on harmfulness of new drugs, to obtain new knowledge' 'To find a cause of a disease' 'To get the correct information regarding something as an illness' 'Search for treatments for diseases as cancer and rabies, Vaccines for prevention Increase productivity of the health sector' 'To assess the weakness of an old treatment' 'To solve problems and provide the right answer' Risks 'Adverse effects from participating in research causing harm or death as in drug trials' 'The harm to placebo group of patients in a drug trial' 'Being cheated due to the ignorance of the public' 'In research done by private institutions, due to signing of agreements there could be problems in withdrawing' 'Research that is not ethical', 'Presenting false research reports' 'Blood samples being used for other things', 'participants are not properly cared for' 'Plundering of genetic resources, selling our resources' 'Abusing people in the third world by developed nations' 'Research on arms development, research that are done with the aim of financial gain and those done in areas that has no benefit for society'

### Who benefits from research?

Most participants (51; 77%) nominated very broad categories of public beneficiaries (*everyone, we, society, public, country, world, humanity*). According to 21 (32%) respondents, *researchers, research institutions and scientists *stood to benefit from research. Other nominations included patients (12; 18%), businessmen, drug manufacturers and companies (9; 14%), health professionals (9; 13%) and policy makers (2; 3%).

### How are research participants selected?

Two themes emerged from half (33) of the study participants' views on this topic:

1. *Depending on the requirements of the research question, suitable/relevant individuals/patients with certain characteristics/features/conditions/diseases, are selected according to inclusion criteria*

2. *According to the sampling method of the research, a random sample or if possible all relevant individuals/patients will be selected*

A further 6(9%) participants held the view that *participants were selected from individuals giving consent or volunteering by themselves or by choice of participant*.

Among the 30 (45%) participants who had previously taken part in research, the following descriptions were given of the type of study in which they had been involved: *undergoing investigations, market surveys, health and community surveys*.

Some of the responses are reported below verbatim for clarity

'Those with grater skill in research, specialists, researchers'

'According to the connections of the researcher'

'Those with greater knowledge'

'Those with the dedication'

'Ask for those willing to participate after calling people to gathering in committees'

'According to patient's choice'

'Individual thought to be suitable by the researchers, that is willing to make a sacrifice, able to conduct one self in society'

'Level of education, social status, job, according to the problems, ability to answer questions'

### Willingness to take part in research

The overwhelming majority (59; 89%) expressed their willingness to participate in future research while five (8%) said they would not and two (3%) were undecided.

While 17 (26%) participants said they would not consult anyone before deciding whether to take part in research, the majority (48; 73%) indicated that they would discuss with someone before giving consent to take part in research. Thirty six (75%) of this group said they would consult their *family and friends*, 16 (33%) would consult *a knowledgeable person on the matter, and 14 (29%) would consult a researcher or research assistant*. One (2%) participant thought consulting *superiors *would prove to be the most appropriate.

### Types of research willing or unwilling to join

Table 7 shows types of research that the participants were willing or unwilling to join.

**Table 7:** Types of research willing or unwilling to join.

Concept	Themes	Supporting Quotes
**Types of research willing to join**	Research that involves donating tissue samples from body including blood (35.8%) Health research (15%), questionnaire research (11%) Research that benefits society 11%) Do not wish to segregate research or not particular about the type of research (18.9%)	'Those that answer the problems faced in life' 'Research that is of benefit to the country and public Education research' 'Research on rehabilitation of disabled children'

**Types of research unwilling to join**	Involving testing of a new drug or a treatment (36%) Invasive/interventional research with instrumentation/research where there is introduction of something in to the body or taken internally (26%) Done only for the benefit of the researcher/of no benefit to society or detrimental to the country (13%)	'If have to go to some other place' 'Participating in medical tests' 'Things that go by the name research but that are not research' 'Research about extremely personal issues' 'Research of an unfamiliar field'

### Information needed for decision making

Two strategies were adopted to examine this aspect. Initially participants' views were sought through open ended questions and their responses were noted verbatim. Secondly, participants were given a pre-defined checklist of information pertaining to the decision to take part in research and asked to note whether each of these was important to them in making the decision to participate in research.

Table 8 lists participants' responses to the open ended question. Table 9 shows their responses to the pre-defined checklist.

**Table 8:** Participants' responses to the open ended questions on important information they wish to have to decide to participate in research.

Objectives of the Research/Reason/Importance/Relevance	27 (43%)
What is the research/Type/Nature/Field	23 (37%)
Potential benefits/risks to society	19 (30%)
Potential benefits/risks to participant	19 (30%)
Credibility & legitimacy of Researchers/Institutions	14 (22%)
What is expected of participant/participation related info/Why selected	11 (18%)
Expected outcome/Protocol rationale/how scientific	10 (16%)
How results would be/should be used	9 (14%)
Methodology	8 (12%)
Confidentiality	3 (5%)
Can withdraw anytime	2 (3%)
Ethical issues/intentions good or bad/information source	2 (3%)

**Table 9:** Participants' responses to the pre-designed checklist on important information they wish to have to decide to participate in research.

	Yes (%)	No (%)	Not sure (%)
1. Description of the project			
1) verbally	62(94%)	3(5%)	1(2%)
2) in writing	59(89%)	6(9%)	1(2%)
2. Who are the researchers?	56(85%)	8(12%)	2(3%)
3. Who is funding?	37(56%)	28(42%)	1(2%)
4. Why have you been selected?	52(79%)	13(20%)	1(2%)
5. It is compulsory to take part?	54(81.8%)	9(14%)	3(5%)
6. A statement assuring that declining to take part will not affect medical care	49(74%)	15(23%)	2(3%)
7. A statement stating that even if they agree to take part it is possible to leave the research at any time	56(85%)	9(14%)	1(2%)
8. What will they have to do as research participants?	64(97%)	1(2%)	1(2%)
9. Informing that they are free not to answer any question or not do thing if they as so wish?	56(85%)	8(12%)	2(3%)
10. Are there any risks involved? If so what are they?	60(91%)	5(8%)	1(2%)
11. Will the information given be confidential?	58(88%)	7(11%)	1(2%)
12. Contact details of main researchers?	55(83%)	10(15%)	1(2%)
13. What are the possible benefits 1)To me?(missing data-1)	6(85%)	8(12%)	1(2%)
2) Wider benefits if any?	65(99%)	0(0%)	1(2%)

Table 8 shows that, unprompted, participants nominated important points of information on research, which appeared as pre-defined items in Table 9. Most participants agreed readily when prompted that the information was important.

An overwhelming 56 (85%) noted that they require a time gap between receiving the information leaflet to taking a decision and notifying the researcher of their decision. Only 9 (13.6%) respondents said that they would not require a time gap.

The majority (61; 92%) were aware that they were participating in a research project right now.

## Discussion

The results show that with or without prior participation in research, participants in this study had a reasonable understanding of research, including the following core information: what is research, why research is conducted, why members of the public are selected, whether the outcome of participation is beneficial or may bring harm and whether the decision to participate is affected by knowledge about research and researchers. The 'reasonable' understanding mentioned above was deemed so after considering the fact that most participants indicated an understanding about the core issues concerning participation in research.

As indicated from some studies conducted in Africa, public will participate in research according to the circumstances regardless of literacy in the absence of other care provision alternatives [15]. Also, our study results indicate that almost 30% of participants were illiterate about the meaning of research. It is in this context that the public knowledge and awareness about core reasons for participation in research presented in this study is important.

In spite of the general understanding of the participants on core ideas about research, a small number of participants believed that research meant laboratory tests ordered by doctors and that the words test and research were similar in meaning. As shown in findings of this study, participants seem to be reluctant to take part in drug research or intervention studies. This is an important finding in view of more and more new drug related clinical trials taking place in developing country settings. But, in answer to a different question in the study, majority of participants expressed willingness to take part in future research, which may mean that although drug trials and interventions are perceived to be harmful in some sense, people are keen to take part in other areas of research perceived to be less harmful. The reasons for people perceiving drug research and interventions to be harmful are areas that merit further exploration.

The findings show that a decision about taking part in research is not only dependent on the knowledge and education of the participant but also on the views of their immediate social network of family and friends. This reflects on the strong family structure inherent in Sri Lankan society which is also common to other countries and cultures in the South Asian region [25]. Within this backdrop, it raises an important point for researchers to address when designing studies and obtaining informed consent in the future.

Majority of health and other research is being conducted by various government agencies which include universities, research institutions and others in Sri Lanka. But, in this study it appears that participants had only a vague idea about government being an agency conducting research. Although the majority had indicated health professionals and scientists to be the sort of people conducting research, they had not observed a link between these professionals and the government, which employs the majority of researchers. This raises questions about public opinion and lack of awareness about government involvement in research in the Sri Lankan context.

## Conclusions

This paper stands to add a perspective from a developing Asian country to the existing literature and debate about the issue of public understanding of research. As Sri Lanka has higher health indicators and a better developed health system and infrastructure than most developing countries in Africa and Asia coupled with a high literacy rate [26], it is important to bring out what its people think about health research. The attitudes and beliefs expressed by them may help to shape future and ongoing research in other developing countries.

The results demonstrate that the majority were supportive of health research and all participants confirmed that research is beneficial to the welfare of the society. Contrary to academic assumption that the public is averse to taking part in research [4], our sample expressed willingness to take part, even in research that requires taking body tissue samples. This is an encouraging sign. Given this good will, it is essential that undue exploitation of public trust and support for research should be prevented by adhering to sound ethical principles.

## Limitations

Participants were drawn from diverse educational, socio-cultural and professional backgrounds, with and without previous research experience. Therefore, differences between subgroups could not be specifically investigated. However, the aim was to capture a wide range of views and understanding on research and consent. Although the findings cannot be generalised to the whole population, the majority of participants appear to be educated and knowledgeable about basic health research and held positive attitudes. They will continue to be supportive of sound ethical research agendas. This work has also opened up the scope for more research in these areas expanding some of the positive as well negative perceptions revealed in the study.

## Competing interests

The authors declare that they have no competing interests.

## Authors' contributions

AS proposed the initial conceptual framework for the research and was responsible for overall conduct of the project. AS, SS, SH, MP and JM were involved in the protocol design, writing and editing the manuscript. SS, SH, ML, MA, CS, KM, KS contributed to the modification of the protocol, collection and analysis of data including the statistical analysis. KM and KS were involved in the reanalysis of data. AS prepared, the first draft and AS, CS, SS wrote the second draft. All authors were involved in subsequent editing and agreed on the final version. CS and AS further edited the paper as required by reviewers.

## Appendix

### The interview schedule for general public who have not participated in research previously

*In this study we are mainly interested in healthcare research. So can you tell me*

1. What does the word "research" mean to you?

2. Is there any other word for research?

3. In your opinion what sort of people will do research?

4. Why do you think they do healthcare research? What is the purpose?

5. Is it a good thing to do research? Can you give an example?

6. Are there any bad things about research? Can you give an example

7. Who do you think benefits from research?

8. How do people get selected or invited for research?

9. Have you ever participated in research? If yes, can you tell me about it?

10. If you are asked to participate in a research in the future will you participate?

11. Would you like to discuss it with someone else before you agree?If yes, who are they//who would that be?

12. What are the types of research that you would

• Like to participate in

• Not Like to participate in

13. What information would you like to have about the research that would help you to decide to or not to participate in research?

14. Which of the following information would you think is/are important for the decision?

CHECKLIST-A →

• Description of the project reflecting what it is about

Verbally

In writing

• Who are the researchers?

• Who provided the funds?

• Why have you been selected?

• Is it compulsory to take part (is it entirely voluntary)?

• A statement assuring that in the case of declining to take part, it will have no affect on the medical care that they receive or any other affect on them

• A statement stating that even if they agree to take part it is possible to leave the research at any point

• What will they have to do as research participants?

• Informing that they are free not to answer any questions or not to do things if they as so wish

• Are there any risks involved? If so what are they?

• Will the information given be confidential?

• Contact details of main researchers

• What are the possible benefits?

To me

& wider benefits if any

• Any other

15. Do you need to have a gap between giving the information leaflet and in deciding whether to participate?

16. Do you realize that you are engaged at the moment is also a research?

## Pre-publication history

The pre-publication history for this paper can be accessed here:

http://www.biomedcentral.com/1472-6939/11/7/prepub
